# The financial impact of NIH's indirect cost cap on higher education research

**DOI:** 10.1093/haschl/qxaf094

**Published:** 2025-05-02

**Authors:** Eric W Ford, Timothy R Huerta

**Affiliations:** School of Public Health, University of Alabama Birmingham, 1720 2nd Avenue South, Birmingham, AL 35294, United States; Department of Family and Community Medicine, The Ohio State University College of Medicine, The Ohio State University Wexner Medical Center, Columbus, OH 43210, United States

**Keywords:** National Institutes of Health funding, federal funding, research policy

## Abstract

On February 7, 2025, the National Institutes of Health (NIH) announced a policy setting all facilities and administrative (F&A) rates at 15% for grants awarded to Institutions of Higher Education (IHEs), replacing negotiated rates that often exceeded 50%. This change poses significant financial challenges for IHEs. On April 4, 2025, a permanent injunction was issued, preventing the NIH from implementing the new policy. To quantify the projected financial impact of the NIH's new F&A cap on U.S. IHEs and assess its broader implications for research sustainability. We analyzed NIH funding data from the NIH RePORTER database for Fiscal Year 2024 (FY2024). Institutions were categorized by public vs private status, and revenue losses under the new policy were calculated. State-level impacts were also assessed. NIH's F&A cap is projected to reduce IHE funding by $5.24B in FY2025. Public universities would lose $2.99B, while private universities face a $2.25B reduction. States with high research expenditures and historically high F&A rates would experience the greatest financial strains. The policy may weaken U.S. research capacity, disproportionately impact public institutions, and shift funding reliance toward state legislatures and private partnerships, with long-term consequences for biomedical innovation.

## Teaser

The National Institutes of Health's (NIH) decision to impose a 15% flat rate on facilities and administrative (F&A) costs for grants to institutions of higher education has triggered widespread financial concerns, particularly for public universities that rely heavily on these reimbursements. This policy change threatens to reduce institutional research funding by nearly $5 billion annually, forcing universities to reassess hiring, infrastructure investments, and research priorities. Public institutions are projected to bear the brunt of these reductions, potentially exacerbating disparities in research funding across states. In response to mounting legal challenges, a federal court issued an indefinite restraining order on February 21st and a permanent injunction on April 4, 2025, halting implementation of the policy. The legal pause grants universities time to mobilize legislative and advocacy efforts, but uncertainty remains about long-term research sustainability. The analysis herein quantifies the financial impact of the policy, explores institutional responses, and examines the broader implications for U.S. research competitiveness. Will this funding shift force institutions to rely more on private partnerships, or can policy interventions mitigate the damage? Read on to explore the financial, legal, and strategic consequences of NIH's contested policy change.

## Introduction

Research projects' budgeting commonly includes the direct costs necessary to conduct studies (eg, salaries, travel, equipment) and indirect costs (IDC), known as facilities and administrative (F&A) costs.^1^ The IDC cover joint expenses at the institutional level that are critical to conduct research but cannot be itemized for each project (eg, utilities, maintenance of facilities, information technology, administrative support).^1^

The National Institutes of Health (NIH) announced a policy standardizing F&A rates at a universal “flat” 15% for grants awarded to Institutions of Higher Education (IHEs) on February 7th, 2025.^2^ The guidance replaced institutions' individually negotiated F&A rates that often exceeded 50%. As of February 21, 2025,^3^ the rule change was blocked and Massachusetts's judge has issued a permanent injunction on the order's implementation on April 4, 2025.^4^ Later that week (April 8, 2025), the Trump Administration appealed the injunction to a higher U.S. Court of Appeals.^4^

The policy change, if implemented, is expected to significantly impact the financial landscape at research focused IHEs and the communities they inhabit. Every dollar of grant funding from the NIH is estimated to generate approximately $2.56 in local economic activity.^5^ Therefore, the financial implications of the proposed policy are likely far more impactful beyond the direct reductions proposed.

Using data extracted from the NIH Reporter's 2024 fiscal year (FY2024) database, this article quantifies the expected revenue losses across public and private universities at the state-level using both a “flat” and a “capitated” rate. If a strict reading of the notice is implemented with a “flat” 15% rate for all grants, some existing awards with rates lower than 15% would be increased—potentially offsetting some losses. A more conservative interpretation of the notice is that grants will be “capped” at 15% and those already below that threshold will remain at the lower level. Both scenarios financial impacts are estimated, and the broader implications for research funding and sustainability are discussed.

## Background on NIH grant programs

Historically, the NIH solicits research ideas through structured mechanisms such as Requests for Applications and Requests for Proposals.^6^ These outline NIH priorities set by governmental policy and strategic goals. Applications undergo rigorous peer-review to ensure funding goes to projects that substantially advance scientific discovery and address critical health issues. Thus, researchers must consistently align their proposals with NIH priorities and compete vigorously for funding.

F&A costs support essential shared resources not directly allocable to specific projects, including utilities, IT infrastructure, administrative oversight, and compliance activities. For context, private-sector industries such as accounting, law, and consulting commonly charge overhead rates around 200%-300% of an employee's direct hourly salary. In comparison, universities typically negotiate F&A rates between 30% and 60%, suggesting higher education institutions receive relatively modest overhead reimbursements compared with industry standards.^7^ While the NIH notice cites foundations' lower F&A rates as a rationale for the proposed cap, it is important to recognize that foundations often permit certain indirect expenses to be budgeted directly, mitigating some differences in stated overhead rates.

University F&A reimbursements also cover ethical oversight and regulatory compliance, including Institutional Review Board (IRB) reviews, informed consent processes, ongoing ethical training, and monitoring research conduct.^8^ Regulatory compliance encompasses federally mandated requirements such as data security, animal welfare, biosafety, and financial audits. Collectively, these oversight and compliance activities ensure institutional accountability, safeguard research integrity, and maintain public trust in university-led research endeavors.

## Methods

To assess the financial impact of the NIH's new F&A policy, we exported data from the NIH RePORTER for all 50 states, capturing awards for FY2024 for Institutions of Higher Education (IHEs).^9,10^ The NIH RePORTER includes the intuitions' names, each grant's direct and indirect amounts awarded, and F&A rates. Institutions receive grants through multiple schools, colleges, and centers, creating some ambiguity when trying to identify their entire portfolio. First, data were sorted by zip code and name. While grants from one IHE might go in under different unit names (ie, “IHE_Name” plus Medicine or “IHE_Name” plus Public Health), the submitting authority generally uses the same zip code with few IHEs sharing a code. Both Excel's fuzzy matching algorithm and visual inspections were used to create single entities. We matched the NIH RePORTER data with the National Center for Science and Engineering Statistics survey from 2024 which includes the institutions' ownership type (eg, public vs private) using the institutions' name and contact office zip codes.

The total lost revenues for *each* grant were calculated. For grants receiving F&A in excess of 15%, the new F&A payment was calculated. For grants with an F&A below 15%, two calculations were made—one raising it to the 15% “flat” rate and second leaving it as previously negotiated for a “capitated” rate. All new calculations were aggregated up to the state-level for both private and public institutions. Both the capitated and flat models are presented, but the more conservative estimate is used for discussion purposes. In addition, the per capita impact of the change in funding level is calculated to create a more granular assessment of state-level impacts.

## Findings

Table 1 presents the NIH's FY2024 funding on a state-by-state basis overall and stratified by both private vs public institutions and direct vs F&A allocations (See Table 1). In addition, the recalibrated F&A amounts are presented. The data reveal that the average F&A rate for IHEs in FY2024 was approximately 37.2%, with some institutions exceeding 50%. Applying a capitated rate of 15% or lower, the NIH's new F&A model is projected to reduce overall funding to IHEs by approximately $5.24 billion in FY2025 (unadjusted for inflation), with public universities losing an estimated $2.99 billion and private universities facing a reduction of $2.25 billion. Applying a uniform, flat 15% rate to every grant, the NIH's new F&A cap is projected to reduce overall funding to IHEs by approximately $4.96 billion in FY2025 (unadjusted for inflation), with public universities losing an estimated $3.35 billion and private universities facing a reduction of $1.61 billion (see Appendix A).

**Table 1.** NIH funding distribution by state before and after policy change^a^.

	Statewide summary			15% F&A capped			
State	Total direct cost	Total F&A costs	F&A rate	Total F&A costs	F&A reduction	Effective F&A rate	Per capita loss
AK	$7952	$3241	40.8%	$1058	$2184	13.3%	$2.95
AL	$328 705	$105 895	32.2%	$45 825	$60 070	13.9%	$11.65
AR	$78 839	$28 647	36.3%	$11 060	$17 587	14.0%	$5.69
AZ	$231 483	$77 732	33.6%	$31 416	$46 316	13.6%	$6.11
CA	$3 166 393	$1 168 945	36.9%	$424 624	$744 320	13.4%	$18.88
CO	$379 264	$133 749	35.3%	$52 454	$81 295	13.8%	$13.65
CT	$585 794	$255 357	43.6%	$78 828	$176 528	13.5%	$48.03
DC	$129 487	$40 534	31.3%	$17 654	$22 880	13.6%	$32.58
DE	$57 781	$22 028	38.1%	$8221	$13 806	14.2%	$13.13
FL	$619 298	$207 705	33.5%	$88 265	$119 441	14.3%	$5.11
GA	$596 184	$219 672	36.8%	$83 151	$136 521	13.9%	$12.21
GU	$2805	$1059	37.7%	$421	$638	15.0%	$3.80
HI	$65 543	$27 073	41.3%	$9475	$17 599	14.5%	$12.17
IA	$159 678	$65 417	41.0%	$22 611	$42 806	14.2%	$13.21
ID	$26 123	$7983	30.6%	$3785	$4198	14.5%	$2.10
IL	$896 984	$357 307	39.8%	$123 821	$233 485	13.8%	$18.37
IN	$322 160	$125 109	38.8%	$45 267	$79 842	14.1%	$11.53
KS	$124 451	$45 575	36.6%	$17 737	$27 838	14.3%	$9.37
KY	$191 368	$79 598	41.6%	$27 872	$51 726	14.6%	$11.27
LA	$188 928	$65 603	34.7%	$26 863	$38 740	14.2%	$8.43
MA	$1 023 773	$366 449	35.8%	$140 314	$226 135	13.7%	$31.69
MD	$950 863	$345 483	36.3%	$126 080	$219 403	13.3%	$35.03
ME	$13 396	$4061	30.3%	$1923	$2138	14.4%	$1.52
MI	$780 849	$290 476	37.2%	$107 605	$182 871	13.8%	$18.03
MN	$334 294	$123 874	37.1%	$46 143	$77 731	13.8%	$13.42
MO	$765 026	$258 950	33.8%	$99 884	$159 066	13.1%	$25.47
MS	$66 773	$21 144	31.7%	$9510	$11 634	14.2%	$3.95
MT	$32 437	$9490	29.3%	$4455	$5035	13.7%	$4.43
NC	$1 080 426	$403 200	37.3%	$150 828	$252 372	14.0%	$22.85
ND	$28 116	$8540	30.4%	$4007	$4532	14.3%	$5.69
NE	$109 985	$44 868	40.8%	$16 132	$28 736	14.7%	$14.33
NH	$86 108	$40 370	46.9%	$12 506	$27 865	14.5%	$19.78
NJ	$260 390	$102 535	39.4%	$36 502	$66 033	14.0%	$6.95
NM	$101 812	$37 902	37.2%	$14 348	$23 555	14.1%	$11.06
NV	$28 354	$9197	32.4%	$3995	$5202	14.1%	$1.59
NY	$2 403 095	$926 631	38.6%	$328 421	$598 210	13.7%	$30.11
OH	$573 004	$233 311	40.7%	$80 623	$152 687	14.1%	$12.85
OK	$98 404	$29 988	30.5%	$14 213	$15 775	14.4%	$3.85
OR	$311 485	$101 535	32.6%	$40 322	$61 212	12.9%	$14.33
PA	$1 427 886	$575 886	40.3%	$200 612	$375 274	14.0%	$28.69
PR	$65 711	$18 781	28.6%	$8921	$9860	13.6%	$3.08
RI	$150 885	$51 333	34.0%	$20 842	$30 490	13.8%	$27.41
SC	$206 760	$72 514	35.1%	$28 655	$43 859	13.9%	$8.01
SD	$12 349	$3832	31.0%	$1708	$2123	13.8%	$2.30
TN	$170 597	$60 796	35.6%	$23 831	$36 965	14.0%	$5.11
TX	$1 495 695	$549 447	36.7%	$207 569	$341 878	13.9%	$10.93
UT	$222 062	$82 196	37.0%	$30 956	$51 241	13.9%	$14.63
VA	$298 331	$121 208	40.6%	$41 990	$79 217	14.1%	$8.99
VI	$418	$29	6.8%	$29	$0	6.8%	$-
VT	$44 962	$17 018	37.9%	$6309	$10 710	14.0%	$16.51
WA	$527 672	$186 409	35.3%	$72 934	$113 474	13.8%	$14.26
WI	$436 076	$154 956	35.5%	$57 604	$97 352	13.2%	$16.33
WV	$50 766	$16 433	32.4%	$6892	$9541	13.6%	$5.39
WY	$11 799	$4532	38.4%	$1770	$2762	15.0%	$4.70
Grand Total	$22 329 777	$8 311 600	37.2%	$3 068 842	$5 242 758		

^a^All figures are presented in $1 000's.

**Figure 1.**
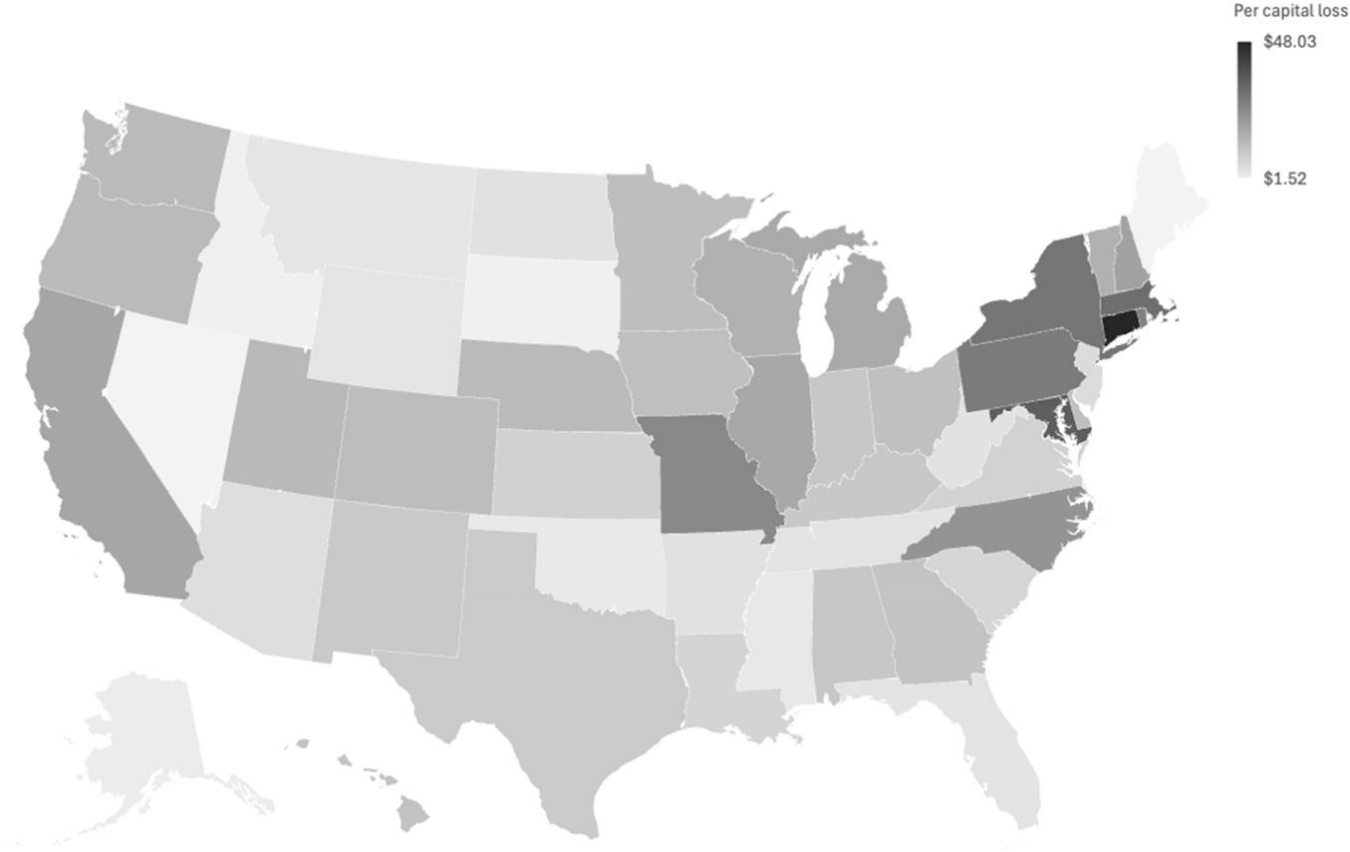
Indirect recovery loss per capita (50 states and the district of Columbia).

The absolute reductions would be disproportionately higher in states with the largest research institutions and in those with the highest effective F&A rates. For example, California, New York, and Pennsylvania would experience the largest absolute funding losses due to the scale of their research enterprises, while states such as New Hampshire, Connecticut, and Kentucky would face the largest proportional reductions due to historically high negotiated F&A rates. On a per capita basis, Connecticut, Maryland, the District of Columbia, and Massachusetts would experience the largest declines in F&A recovery under a 15% capitated system (See Figure 1).

A comparison of public vs private institutions highlights additional differences. Public universities, which rely heavily on state appropriations and tuition revenue, might face significant financial pressures from these reductions, particularly in states where legislative budgets are already strained. Private universities, which often have larger endowments and greater access to philanthropic funding, may be better positioned to adapt to the changes but might still experience notable disruptions to research funding and institutional planning.

## Discussion

The financial impact of NIH's F&A cap extends beyond individual institutions to broader economic considerations at the local, state, and federal levels. Public and private institutions play critical roles in regional economies, supporting job creation, workforce development, and technological innovation. However, public institutions remain particularly vulnerable, as the potential funding reductions might necessitate legislative intervention to preserve essential research and educational functions.

### Legal challenges and the permanent restraining order

The policy's immediate implementation was halted on February 21, 2025, when a federal judge issued a Temporary Restraining Order in response to legal challenges led by 22 state attorneys general and the Association of American Medical Colleges (AAMC). The ruling was modified in April 2025 to be a permanent injunction on the F&A cap reduction while the court reviews its legality and broader implications. This decision has provided a reprieve for research institutions, enabling them to continue receiving negotiated F&A reimbursements while exploring alternative advocacy and policy strategies.

While the injunction delays immediate financial consequences, institutions remain uncertain about long-term NIH funding stability and priorities. Universities may continue lobbying Congress for a legislative resolution or pursuing exemptions for high-cost research programs. However, should the NIH 15% capitation policy ultimately be upheld, the sector will still face significant revenue reductions requiring structural adjustments.

The NIH has an alternative, albeit incremental path to reducing F&A rates. IHEs renegotiate their F&A rates every 3-4 years. The NIH could adopt a policy capping rates at 15% during that evolution. This suggests that IHEs currently in the renegotiation cycle would be impacted first, while those whose reviews fall four years out will at a minimum have longer time to plan.

### Institutional responses and policy adjustments

Extrapolating from FY2024 data, full implementation of the F&A cap would require public and private universities to adjust their budgets significantly downward. Without intervention, universities might be forced to implement hiring and wage freezes, restructure research funding models, and prioritize revenue diversification through state appropriations, private partnerships, and philanthropic fundraising.

### Potential mitigation strategies include

- Phased Implementation: Instead of an abrupt shift, policymakers might consider a gradual transition to lower F&A rates, allowing institutions time to adjust.
- Exemptions for High-Cost Research: Some fields, such as biomedical engineering and clinical trials, incur substantially higher administrative costs and may require modified F&A treatment.
- State-Level Interventions: Legislatures could provide emergency funding or incentives to offset the federal reduction's impact on public universities, but that is unlikely to be a sustainable model.

### Unintended consequences and future research sustainability

Even if the F&A cap is ultimately enacted, the long-term consequences of funding redistribution could reshape the higher education research landscape. Ironically, these federally mandated costs imply that NIH's cap on F&A reimbursement rates may inadvertently compromise institutions' capacity to fulfill required oversight responsibilities. For example, IRB, regulatory compliance, secure data warehousing, and other critical services may shrink, potentially affecting research integrity and safety.

More broadly, the shift might favor private institutions with large endowments, while smaller or emerging research universities could struggle to compete for NIH funding due to reduced overhead recovery. This could lead to increased reliance on industry partnerships that often prioritize commercial applications over foundational scientific inquiry. Additionally, the uncertainty surrounding the policy's future may already be affecting institutional planning. Some universities have begun delaying new research commitments, anticipating a scenario where they must absorb large funding gaps.^11^ This hesitation could slow innovation and reduce the U.S.'s competitive edge in biomedical research.

Moreover, even if the proposed F&A rate reductions do not come to pass, it is likely that NIH’s funding priorities and overall budget allocations will shift, challenging current institutional operating models. Institutions must prepare for potential reallocations of federal research dollars, which could influence funding availability across different fields and research domains. These adjustments may necessitate long-term strategic planning and alternative funding mechanisms to sustain research programs.

## Limitations

The process of identifying IHE in the NIH Reporter is likely to misclassify some awards due to unclear naming or ownership issues. For example, Southern Research Institute is a wholly owned subsidiary of the Alabama System, but not an explicit part of an IHE. Similarly, many IHE originated applications are run through health systems that do not have an obvious affiliation that the matching process would have correctly identified. In both cases, these types of awards were not included herein. The net effect is that the results are likely a conservative or underestimate of the overall impact of F&A reductions.

## Conclusion

The NIH's attempt to capitate F&A reimbursement at 15% has sparked significant financial and legal controversy. While the April 7, 2025, permanent injunction forestalled the reductions, the long-term implications remain uncertain. The policy's suspension allows universities to mobilize advocacy efforts, work with policymakers, and explore alternative funding strategies, but a resolution has yet to be reached.

If the policy is upheld, it could weaken research capacity, disproportionately impact public institutions, and lead to increased dependence on private-sector funding. However, should legal challenges succeed in overturning the policy, universities will likely push for structural reforms in federal research funding to prevent similar disruptions in the future.

Regardless of the policy outcome, NIH's funding levels and strategic priorities are likely to evolve, requiring institutions to adapt to new federal funding landscapes. Universities must consider diversifying funding sources, advocating for sustained research investment, and developing adaptable financial strategies to ensure long-term research viability.

Going forward, research institutions must remain proactive, engaging with legislators and funding agencies to develop sustainable policies that balance fiscal responsibility with the continued advancement of scientific discovery. The outcome of ongoing legal proceedings will be pivotal in determining whether U.S. universities can maintain their role as global leaders in research innovation.

## Supplementary Material

qxaf094_Supplementary_Data
