# Supplementary material for: The financial impact of NIH's indirect cost cap on higher education research
Source: Health Aff Sch. 2025 May 2;3(6):qxaf094. doi: 10.1093/haschl/qxaf094 (PMC12152721; doi:10.1093/haschl/qxaf094)
Supplement: qxaf094_Supplementary_Data [file qxaf094_supplementary_data.zip › Appendix Analysis of NIH FandA Policy Change 20250414 HAS.docx]

Appendix A: Public and Private IHE’s 2024 Direct and Indirect Contract Costs by State

|  | **Public** | | |  | **Private** | | |  | **Statewide Summary** | | |
| --- | --- | --- | --- | --- | --- | --- | --- | --- | --- | --- | --- |
| State | Direct Costs | F&A Costs | State F&A Rate |  | Direct Costs | F&A Costs | State F&A Rate |  | Total Direct Cost | Total F&A Costs | F&A Rate |
| AK | $7,952 | $3,241 | 40.8% |  |  |  |  |  | $7,952 | $3,241 | 40.8% |
| AL | $320,287 | $102,330 | 31.9% |  | $8,417 | $3,565 | 42.4% |  | $328,705 | $105,895 | 32.2% |
| AR | $78,703 | $28,628 | 36.4% |  | $136 | $19 | 14.0% |  | $78,839 | $28,647 | 36.3% |
| AZ | $230,650 | $77,473 | 33.6% |  | $833 | $259 | 31.1% |  | $231,483 | $77,732 | 33.6% |
| CA | $2,306,011 | $833,474 | 36.1% |  | $860,382 | $335,471 | 39.0% |  | $3,166,393 | $1,168,945 | 36.9% |
| CO | $373,713 | $131,608 | 35.2% |  | $5,551 | $2,142 | 38.6% |  | $379,264 | $133,749 | 35.3% |
| CT | $73,489 | $31,631 | 43.0% |  | $512,306 | $223,726 | 43.7% |  | $585,794 | $255,357 | 43.6% |
| DC | $404 | $11 | 2.8% |  | $129,083 | $40,523 | 31.4% |  | $129,487 | $40,534 | 31.3% |
| DE | $57,781 | $22,028 | 38.1% |  |  |  |  |  | $57,781 | $22,028 | 38.1% |
| FL | $450,193 | $151,943 | 33.8% |  | $169,105 | $55,763 | 33.0% |  | $619,298 | $207,705 | 33.5% |
| GA | $162,765 | $64,170 | 39.4% |  | $433,420 | $155,502 | 35.9% |  | $596,184 | $219,672 | 36.8% |
| GU | $2,805 | $1,059 | 37.7% |  |  |  |  |  | $2,805 | $1,059 | 37.7% |
| HI | $65,543 | $27,073 | 41.3% |  |  |  |  |  | $65,543 | $27,073 | 41.3% |
| IA | $158,942 | $65,220 | 41.0% |  | $737 | $197 | 26.8% |  | $159,678 | $65,417 | 41.0% |
| ID | $25,716 | $7,900 | 30.7% |  | $407 | $83 | 20.3% |  | $26,123 | $7,983 | 30.6% |
| IL | $196,088 | $85,248 | 43.5% |  | $700,895 | $272,059 | 38.8% |  | $896,984 | $357,307 | 39.8% |
| IN | $298,813 | $117,227 | 39.2% |  | $23,347 | $7,882 | 33.8% |  | $322,160 | $125,109 | 38.8% |
| KS | $124,451 | $45,575 | 36.6% |  |  |  |  |  | $124,451 | $45,575 | 36.6% |
| KY | $191,068 | $79,469 | 41.6% |  | $300 | $128 | 42.8% |  | $191,368 | $79,598 | 41.6% |
| LA | $90,664 | $29,002 | 32.0% |  | $98,264 | $36,602 | 37.2% |  | $188,928 | $65,603 | 34.7% |
| MA | $196,511 | $82,094 | 41.8% |  | $827,261 | $284,355 | 34.4% |  | $1,023,773 | $366,449 | 35.8% |
| MD | $237,806 | $78,913 | 33.2% |  | $713,057 | $266,570 | 37.4% |  | $950,863 | $345,483 | 36.3% |
| ME | $5,201 | $1,517 | 29.2% |  | $8,194 | $2,544 | 31.0% |  | $13,396 | $4,061 | 30.3% |
| MI | $780,290 | $290,316 | 37.2% |  | $558 | $160 | 28.6% |  | $780,849 | $290,476 | 37.2% |
| MN | $333,919 | $123,844 | 37.1% |  | $375 | $30 | 8.0% |  | $334,294 | $123,874 | 37.1% |
| MO | $66,699 | $28,657 | 43.0% |  | $698,327 | $230,293 | 33.0% |  | $765,026 | $258,950 | 33.8% |
| MS | $58,773 | $21,144 | 36.0% |  | $8,000 | $0 | 0.0% |  | $66,773 | $21,144 | 31.7% |
| MT | $32,347 | $9,483 | 29.3% |  | $90 | $7 | 8.0% |  | $32,437 | $9,490 | 29.3% |
| NC | $508,966 | $177,382 | 34.9% |  | $571,460 | $225,818 | 39.5% |  | $1,080,426 | $403,200 | 37.3% |
| ND | $28,116 | $8,540 | 30.4% |  |  |  |  |  | $28,116 | $8,540 | 30.4% |
| NE | $102,289 | $41,641 | 40.7% |  | $7,696 | $3,227 | 41.9% |  | $109,985 | $44,868 | 40.8% |
| NH | $7,365 | $2,844 | 38.6% |  | $78,743 | $37,526 | 47.7% |  | $86,108 | $40,370 | 46.9% |
| NJ | $208,859 | $81,004 | 38.8% |  | $51,532 | $21,531 | 41.8% |  | $260,390 | $102,535 | 39.4% |
| NM | $101,812 | $37,902 | 37.2% |  |  |  |  |  | $101,812 | $37,902 | 37.2% |
| NV | $28,354 | $9,197 | 32.4% |  |  |  |  |  | $28,354 | $9,197 | 32.4% |
| NY | $679,744 | $239,353 | 35.2% |  | $1,723,351 | $687,278 | 39.9% |  | $2,403,095 | $926,631 | 38.6% |
| OH | $326,832 | $122,726 | 37.6% |  | $246,172 | $110,585 | 44.9% |  | $573,004 | $233,311 | 40.7% |
| OK | $98,104 | $29,862 | 30.4% |  | $300 | $126 | 42.0% |  | $98,404 | $29,988 | 30.5% |
| OR | $310,689 | $101,381 | 32.6% |  | $796 | $153 | 19.2% |  | $311,485 | $101,535 | 32.6% |
| PA | $719,233 | $287,267 | 39.9% |  | $708,653 | $288,619 | 40.7% |  | $1,427,886 | $575,886 | 40.3% |
| PR | $57,859 | $18,351 | 31.7% |  | $7,853 | $430 | 5.5% |  | $65,711 | $18,781 | 28.6% |
| RI | $25,174 | $5,657 | 22.5% |  | $125,711 | $45,676 | 36.3% |  | $150,885 | $51,333 | 34.0% |
| SC | $205,744 | $72,423 | 35.2% |  | $1,016 | $91 | 8.9% |  | $206,760 | $72,514 | 35.1% |
| SD | $12,349 | $3,832 | 31.0% |  |  |  |  |  | $12,349 | $3,832 | 31.0% |
| TN | $53,761 | $21,410 | 39.8% |  | $116,836 | $39,386 | 33.7% |  | $170,597 | $60,796 | 35.6% |
| TX | $1,189,386 | $428,337 | 36.0% |  | $306,309 | $121,110 | 39.5% |  | $1,495,695 | $549,447 | 36.7% |
| UT | $217,215 | $80,126 | 36.9% |  | $4,847 | $2,071 | 42.7% |  | $222,062 | $82,196 | 37.0% |
| VA | $296,614 | $120,905 | 40.8% |  | $1,717 | $303 | 17.6% |  | $298,331 | $121,208 | 40.6% |
| VI | $418 | $29 | 6.8% |  |  |  |  |  | $418 | $29 | 6.8% |
| VT | $44,381 | $16,836 | 37.9% |  | $581 | $182 | 31.3% |  | $44,962 | $17,018 | 37.9% |
| WA | $527,373 | $186,302 | 35.3% |  | $299 | $106 | 35.6% |  | $527,672 | $186,409 | 35.3% |
| WI | $345,842 | $125,528 | 36.3% |  | $90,234 | $29,428 | 32.6% |  | $436,076 | $154,956 | 35.5% |
| WV | $50,766 | $16,433 | 32.4% |  |  |  |  |  | $50,766 | $16,433 | 32.4% |
| WY | $11,799 | $4,532 | 38.4% |  |  |  |  |  | $11,799 | $4,532 | 38.4% |
| Grand Total | $13,086,626 | $4,780,076 | 36.5% |  | $9,243,151 | $3,531,524 | 38.2% |  | $22,329,777 | $8,311,600 | 37.2% |
